# Brachial Artery Pseudoaneurysm as a Complication of Osteochondral Exostosis of the Humerus in Computed Tomography Angiography Images

**DOI:** 10.3390/diagnostics16060941

**Published:** 2026-03-22

**Authors:** Paweł Gać, Michał Wesołowski, Kamil Biedka, Rafał Poręba

**Affiliations:** 1 Department of Environmental Health, Occupational Medicine and Epidemiology, Wroclaw Medical University, 50-345 Wroclaw, Poland; 2 Centre for Diagnostic Imaging, 4th Military Hospital, 50-981 Wroclaw, Poland; 3 Department of Biological Principles of Physical Activity, Wroclaw University of Health and Sport Sciences, 51-612 Wroclaw, Poland

**Keywords:** brachial artery, computed tomography angiography, humerus, osteochondroma, pseudoaneurysm

## Abstract

We present computed tomography angiography images of a rare pseudoaneurysm of the left brachial artery, a complication of idiopathic injury to the artery caused by an osteochondral exostosis of the left humerus. A 22-year-old Caucasian man with no significant medical history was admitted to the emergency department due to sudden, intense pain in his left arm, numbness, and pallor of his left forearm and hand. The patient’s consulting vascular surgeon referred him to the computed tomography (CT) laboratory for a computed tomography angiography (CTA) of the arteries of his left upper limb. In the CTA examination, at the level of the proximal segment of the left brachial artery, an excess of contrast was visualized, measuring up to approximately 1.5 × 1.2 cm in cross-sections and up to approximately 0.7 cm in the craniocaudal dimension. The CTA image was suggestive of a pseudoaneurysm of the left brachial artery. Laterally, the pseudoaneurysm was adjacent to the apex of the imaged osteochondral exostosis on the medial surface of the proximal shaft of the left humerus. A surgical procedure was performed to repair the pseudoaneurysm of the left brachial artery, including removal of the bony exostosis of the left humerus. In summary, relatively common, benign bone lesions can occasionally result in serious vascular complications. CTA is the gold standard for diagnosing these complications.

**Figure 1:**
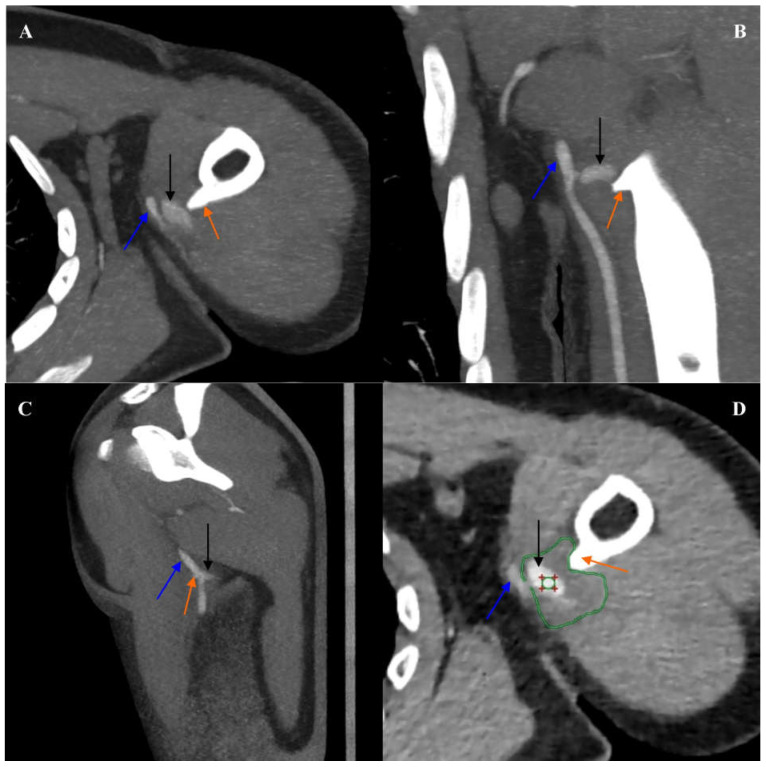
Computed tomography angiography of the left upper limb arteries. Angiographic phase. Multiplanar reconstruction (MPR). (**A**) Axial view. (**B**) Coronal view. (**C**) Sagittal view. (**D**) Axial view. Soft tissue window. The blue arrow indicates the left brachial artery. The black arrow indicates a pseudoaneurysm of the left brachial artery. The orange arrow indicates the osteochondral exostosis of the left humerus. A green circle with red markers for tissue density measurement was placed within the lumen of the left brachial artery pseudoaneurysm. In the soft tissue window, the borders of the thrombosed part of the pseudoaneurysm of the left brachial artery are indicated with a green outline. A 22-year-old Caucasian man with no significant medical history was admitted to the emergency department due to sudden, intense pain in his left arm, numbness, and pallor of his left forearm and hand. The patient’s consulting vascular surgeon referred him to the computed tomography (CT) laboratory for a computed tomography angiography (CTA) of the arteries of his left upper limb. CTA was performed using a 384-slice Siemens Somatom Force CT scanner (Siemens Healthineers, Erlangen, Germany). The study protocol included a topogram, premonitoring, monitoring at the level of the aortic arch with measurement of contrast enhancement of the region of interest (ROI), an angiographic phase triggered when the ROI density of 100 HU was reached, and a delayed phase 60 s after contrast agent administration. The patient was administered 100 ml of iodinated, non-ionic contrast agent (Iomeron 400, Bracco UK Ltd., Oxford, UK) via an automated syringe into the right antecubital vein at an infusion rate of 4.0 mL/s. Axial reconstructions were obtained with slice thicknesses of 5.0 and 0.6 mm. Secondary multiplanar reconstruction (MPR) and volume rendering technique (VRT) reconstruction were performed. In the CTA examination, at the level of the proximal segment of the left brachial artery, lateral to the brachial artery, above the proximal segment of the branching left deep brachial artery, an excess of contrast was visualized, measuring up to approximately 1.5 × 1.2 cm in cross-sections and up to approximately 0.7 cm in the craniocaudal dimension, connecting with the left brachial artery by narrow bands of approximately 0.1 cm and 0.3 cm in width. The CTA image was suggestive of a pseudoaneurysm of the left brachial artery. In the soft tissue window of the CTA, around the above-mentioned excess contrast, a thrombosed component of the pseudoaneurysm was differentiated within the muscles of the left arm, with approximate overall dimensions of 3.4 × 3.2 cm in cross-sections and 2.9 cm in the craniocaudal dimension.

**Figure 2:**
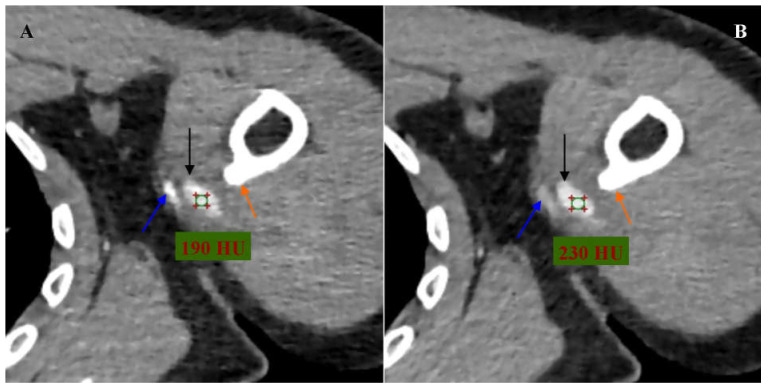
Computed tomography angiography of the left upper limb arteries. (**A**) Angiographic phase. Multiplanar reconstruction (MPR). Axial view. (**B**) Delayed phase. MPR reconstruction. Axial view. The blue arrow indicates the left brachial artery. The black arrow indicates a pseudoaneurysm of the left brachial artery. The orange arrow indicates the osteochondral exostosis of the left humerus. A green circle with red markers for tissue density measurement was placed within the lumen of the left brachial artery pseudoaneurysm. The values indicate the measured lumen density of the pseudoaneurysm of the left brachial artery. The density of the pseudoaneurysm lumen was approximately 190 HU in the angiographic phase and approximately 230 HU in the delayed phase, which indicated active inflow and delayed outflow of contrasted blood into the lumen of the pseudoaneurysm.

**Figure 3:**
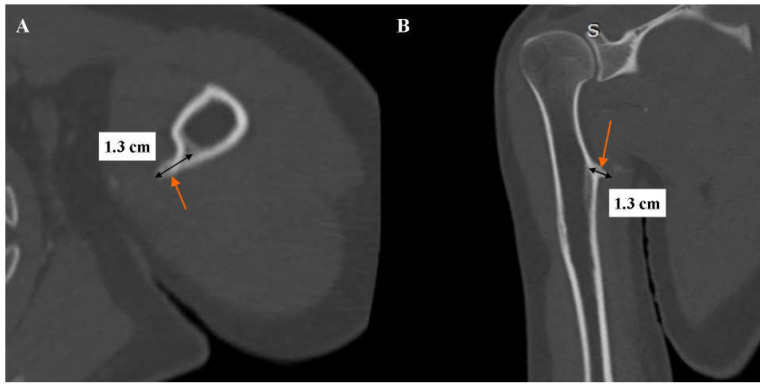
Computed tomography angiography of the left upper limb arteries. Angiographic phase. Multiplanar reconstruction (MPR). Bone window. (**A**) Axial view. (**B**) Oblique view in the long axis of the left humerus. The orange arrow indicates the osteochondral exostosis of the left humerus. The length of the osteochondral exostosis of the left humerus was measured with a black linear marker. The values indicate the measured length of the osteochondral exostosis of the left humerus. Laterally, the pseudoaneurysm was adjacent to the apex of the imaged osteochondral exostosis on the medial surface of the proximal shaft of the left humerus. The measured length of the osteochondral exostosis was approximately 1.3 cm.

**Figure 4:**
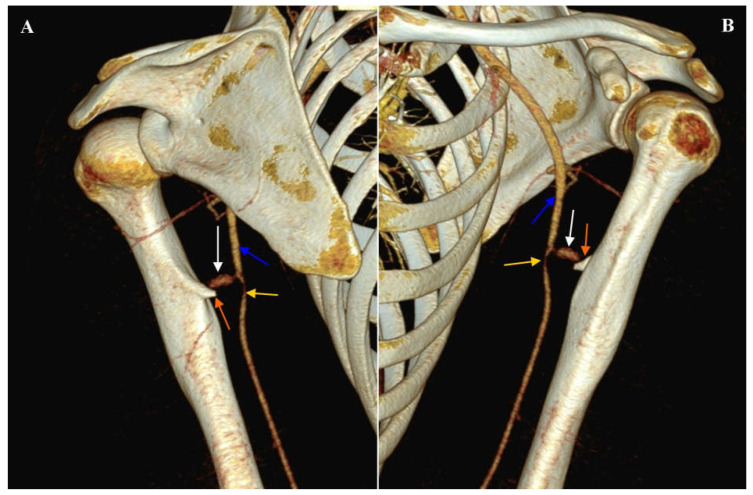
Computed tomography angiography of the left upper limb arteries. Angiographic phase. Volume rendering technique (VRT) reconstruction. (**A**) Posterior view. (**B**) Anterior view. The blue arrow indicates the left brachial artery. The white arrow indicates a pseudoaneurysm of the left brachial artery. The orange arrow indicates the osteochondral exostosis of the left humerus. The yellow arrow indicates narrowing of the left brachial artery caused by compression of the thrombosed part of the pseudoaneurysm. Immediately below the level of the pseudoaneurysm, modeling and compression of the left brachial artery by the thrombosed part of the pseudoaneurysm was observed (over a length of approximately 3.0 cm), with a reduction in the lumen of the artery by 50–70%. Following the necessary qualification tests and patient preparation, a surgical procedure was performed to repair the pseudoaneurysm of the left brachial artery, including removal of the bony exostosis of the left humerus. The removed tissue was submitted for histopathological examination, which confirmed the tissue-related nature of the pathology. The surgical procedure and the early postoperative period were uneventful. The occurrence of osteochondral exostosis on most bones has been described in the literature. Osteochondral exostoses are most observed on the epiphysis of long bones, including the distal femur and proximal tibia, as well as in areas such as the scapula, clavicle, pelvis, vertebrae, and ribs [1,2]. Osteochondral exostosis is usually asymptomatic and detected during imaging studies for other medical reasons. It may present as a palpable bony nodule, but the presence of additional symptoms is primarily related to mechanical pressure on adjacent structures. Possible complications include bursitis, malignant transformation, tingling and numbness associated with nerve compression [3], as well as vascular complications such as thrombosis and vessel perforation, arterial thromboembolic events, and the formation of pseudoaneurysms [4]. In summary, relatively common, benign bone lesions can occasionally result in serious vascular complications. CTA is the gold standard for diagnosing these complications.

## Data Availability

No new data were created.

## Abbreviations

The following abbreviations are used in this manuscript:

CT	computed tomography
CTA	computed tomography angiography
HU	Hounsfield unit
MPR	multiplanar reconstruction
VRT	volume rendering technique
